# Utility of RT-PCR versus electronic track and trace system for pre-procedural COVID-19 screening- a retrospective cohort study

**DOI:** 10.1016/j.heliyon.2023.e15379

**Published:** 2023-04-11

**Authors:** Ammar Chapra, Zohaib Yousaf, Merlin Marry Thomas, Ahmed Ali A Al-Mohammed, Hisham Abdelaleem A Ahmed, Mansoor Hameed

**Affiliations:** a Department of Medicine, Hamad Medical Corporation, Doha, Qatar; b Department of Chest, Hamad Medical Corporation, Doha, Qatar; c Weill Cornell Medicine, Doha, Qatar

**Keywords:** COVID-19, SARS-COV-2, Reverse transcription-polymerase chain reaction, Pre-procedural screening, Contact tracing

## Abstract

**Background and aims:**

COVID-19 has disrupted the patient workflow in all healthcare settings. Procedures such as bronchoscopy and spirometry require additional pre-procedure screening for SARS-CoV-2. However, there is uncertainty regarding the utility of this universal pre-procedure screening. The State of Qatar has a robust contact tracing system in place in the form of the mobile application ‘Ehteraz.’ This study assesses the utility of various pre-procedural screening measures in asymptomatic patients and generate recommendations for any potential improvement in the workflow.

**Methods:**

This is a cross-sectional study of asymptomatic patients who had SARS-CoV-2 RT-PCR screening performed before bronchoscopy or lung function testing scheduled on an elective basis. Descriptive statistics were used to summarize and determine the sample characteristics. The rate of the positive PCR test result was subsequently calculated.

**Results:**

Two patients (0.34%) tested positive for COVID-19 on their pre-procedural screen. Four patients (0.68%) had an inconclusive result.

**Conclusion:**

The positivity rate of SARS-CoV-2 RT-PCR is extremely low in asymptomatic individuals screened before bronchoscopy and spirometry. The authors recommend pre-procedural symptom and electronic application-based contact screening instead of universal pre-procedural SARS-CoV-2 RT-PCR for screening asymptomatic individuals.

## Highlights

• The COVID-19 pandemic has resulted in mandatory pre-procedural screening tests.
• Mobile applications can be used to identify high-risk individuals who should be tested.
• Our results show a positivity rate of less than 1% in asymptomatic patients.
• We recommend not to apply universal COVID-19 screening tests.
• We recommend to screen with COVID-19 symptoms pre-procedurally.

## Introduction

COVID-19 is a global crisis that has brought novel economic, healthcare, social, and administrative challenges. The pandemic has disrupted the well-established healthcare provision pathways, requiring restructuring and introducing inefficiencies in the workflow. The primary aim of these changes is to curtail the transmission of COVID-19 in healthcare settings, thereby adding a layer of fiscal, temporal and procedural difficulties and redundancies.

The primary mode of transmission of COVID-19 is airborne or through respiratory droplets. However, the risk of transmission varies with the prevalence of the disease in the community and the patient's characteristics. Bronchoscopy and pulmonary function testing are essential tools in monitoring respiratory diseases. Bronchoscopy is an aerosol-generating procedure (AGP) that may lead to a higher risk of transmission via the airborne route [1]. Pulmonary function testing could represent a potential avenue for COVID-19 transmission due to the potential for coughing and subsequent droplet formation.

Due to the aerosol generation risks, these elective procedures were deferred universally during the early phase of the pandemic [2]. There is an anticipated increase in demand for these procedures in the post-COVID-19 era due to COVID-19 related pulmonary complications and the backlogs of procedures for non-COVID-19 related respiratory diseases. Respiratory societies like the American thoracic society (ATS), European respiratory society (ERS), and the British thoracic society (BTS) have published specific position statements and recommendations for such procedures [[3], [4], [5]], mainly recommending COVID-19 testing before bronchoscopy and pulmonary function tests irrespective of the presence of symptoms attributed to COVID-19. Additional precautionary measures such as personal protective equipment (PPE) for protecting patients and staff are also used during these procedures.

There is no consensus on the methodology of pre-procedural SARS-CoV-2 screening across various centers. However, most centers used the universal pre-procedural screening with various biochemical assays, including Reverse Transcription-Polymerase Chain Reaction (RT-PCR), with a low diagnostic yield [[6], [7], [8], [9]]. Another bone of contention is the varying sensitivity of various screening tests available for the detection of SARS-CoV-2. The most utilized screening tool is the RT-PCR test, which has a variable sensitivity [10].

Qatar implemented a unique two-pronged national screening approach for COVID-19 at all public places, including medical facilities. A temperature check was performed for all visitors at our hospital using non-contact infrared thermometers or a thermal imaging system. This was followed by a color-coded QR-code check of the national track and trace application “Ehteraz."

In early March 2020, a change in the institutional protocol at Hamad Medical Corporation in Qatar, for aerosol-generating procedures (AGP) made it mandatory for patients to be tested negative for the SARS-CoV-2 virus before any AGPs using the RT-PCR. As a result, all patients underwent pre-procedural testing for COVID-19 regardless of their symptoms. Hamad Medical Corporation is the largest center for interventional pulmonology in the country and one of the largest in the region. Our study aims to evaluate the diagnostic yield of pre-procedural COVID-19 screening before AGPs like bronchoscopy and pulmonary function testing. This study would establish the rate of COVID-19 positivity in asymptomatic patients with a no-contact/low-risk (green) status on the electronic contact-tracing app before bronchoscopy or pulmonary function testing more accurately. In turn, the results may help improve workflow pathways by facilitating timely procedures with less onerous and cumbersome pre-procedural screening and determining the real-world added financial burden of the current screening procedures compared to screening by electronic contact tracing app in conjunction with symptoms and temperature checks.

## Methods

### Study methodology

This is a single-center retrospective cohort study involving retrospective review of patient records of asymptomatic individuals who underwent pre-procedural SARS-CoV-2 screening before bronchoscopy or pulmonary function testing scheduled on an elective basis. The charts were reviewed from March 1, 2020, to November 14, 2020. These dates coincide with the first wave of COVID-19 in the State of Qatar, including the peak prevalence of infections recorded throughout the year.

### Hypothesis

We hypothesized that universal screening for COVID-19 using the RT-PCR was unnecessary in an already overburdened health care system during the COVID-19 pandemic and that pre-procedural screening using the novel electronic track and trace system ‘Ehteraz’ along-with symptom screening would be sufficient. Additionally the study aimed to assess the incidence of asymptomatic positivity rate of COVID-19.

### Inclusion criteria

All patients above the age of 14, who were asymptomatic, and undergoing bronchoscopy or lung function testing electively from outpatient clinics were included in the study.

### Exclusion criteria

Anyone below the age of 14, or who had symptoms (as defined in Appendix 1), or who underwent the above mentioned procedures in the in-patient setting were excluded from the study.

A total of 665 asymptomatic patients were referred for bronchoscopy or spirometry during the mentioned time period all of whom were screened initially. 79 patients were subsequently excluded based on the inclusion criteria. Hence, the records of a total of 586 patients were retrospectively reviewed and included in the final analysis.

Patients were screened for symptoms (Appendix 1) before their procedures in an outpatient setting, and the absence of symptoms was documented while ordering the COVID-19 PCR test. In most cases, the pre-procedural screening was done within the last 48 h preceding the procedure but was acceptable if done within 72 h. Symptoms were also rechecked on the day of RT-PCR screening and the day of the procedure.

At every visit, the Ehteraz application Q.R. color code and temperature of the patient were also checked. “Ehteraz” means “precaution” in Arabic. This application is a mode of electronic track and trace system implemented nationally since the early pandemic. The system comprises an IOS™ and Android™ based mobile application, “Ehteraz.” The app is also connected to a live database with the results of all individuals who had a screening test for COVID-19 performed at any testing center across the country. The Ehteraz application displays a colored Q.R. code. Green color, indicating a subject with no contact to COVID-19 and at low-risk for COVID-19; yellow color for anyone in quarantine; grey color for individuals with contact to a COVID-19 case and high probability of infection and awaiting their test results and red color for those with an active COVID-19 infection. Any Ehteraz status other than green or a hyperthermic individual would be referred to a fever clinic for COVID-19 testing.

COVID-19 testing protocol.

Patients underwent pre-procedural screening via the combined nasopharyngeal/oropharyngeal swab. Subsequent testing of the SARS‐CoV‐2 reverse transcription‐polymerase chain reaction (RT‐PCR) tests was carried out using the Thermo Fisher TaqPath™ COVID-19 RT-PCR kit (Thermo Fisher, USA), the Roche Cobas® 6800 SARS-CoV2 test (Roche, Switzerland), and the Xpert® Xpress SARS-CoV-2 (Cepheid, USA). The details of the swabbing technique are outlined in Appendix 2. The results of the PCR test were reported as one of the following three categories (positive, negative, or inconclusive) based on the manufacture guidance (Appendix 3).

### Procedures

The procedures which were performed included.

1) Bronchoscopic procedures: flexible fiberoptic bronchoscopy (FOB) in a standard negative pressure bronchoscopy room, interventional bronchoscopic techniques via rigid bronchoscopy, and EBUS guided‐transbronchial needle aspiration (EBUS‐TBNA).
2) Pulmonary function testing, including spirometry.

Healthcare workers performed all procedures with full PPE detailed in Appendix 4 per Centers for Disease Control and Prevention (CDC) guidelines for protection during aerosol-generating procedures even though the patients were tested negative.

### Outcome

The primary outcome of our study was to determine the COVID-19 RT-PCR screening test's positivity in asymptomatic patients with a status of “no contact” (green) on the electronic COVID-19 contact tracing app Ehteraz.

### Covariates

Patient data were extracted from electronic medical records, including demographics, type of screening test utilized for COVID-19, screening test result, presence of symptoms of COVID-19, type of procedure performed, and the primary indication for the procedure.

### Statistical analysis

Descriptive statistics were used to summarize and determine the sample characteristics and distribution of various parameters related to demographic, diagnostic, clinical features, and related outcome measures. The normally distributed data and results were reported with mean and standard deviation (S.D.); the remaining results were reported with median and interquartile range (IQR). Categorical data were summarized using frequencies and percentages. The statistical analysis was done using Jamovi 1.2.27. The Jamovi project (2020). jamovi. (Version 1.2) [Computer Software]. Retrieved from https://www.jamovi.org.

#### Study approval

The study was conducted after review and approval by the Medical Research Center (MRC) at Hamad Medical Corporation, Doha, Qatar under ID MRC- 01- 20- 1124 on November 25th, 2020.

The data was collected retrospectively from patient charts and anonymized before analysis. Since this was a retrospective chart review, no informed consent was required as per the policy of the Medical Research Center (MRC) at Hamad Medical Corporation, Doha, Qatar.

The study was conducted in full conformance with principles of the “Declaration of Helsinki,” Good Clinical Practice (GCP), and within the laws and regulations of the Ministry of Public Health in the State of Qatar.

#### Results

A total of 586 patients were included in the analysis. The median age was 39 (29–52). 351 (60%) of patients were male, while the remaining 235 (40%) were females. Their demographic data is detailed in (Table 1). None of the 586 patients reported having any symptoms of COVID-19 before them undergoing the screening test. All the patients had an Ehteraz status of ‘green’ or no-contact and had the combined nasopharyngeal/oropharyngeal RT-PCR test as the type of screening used.

**Table 1:** Demographic information of patients referred for bronchoscopic procedures and lung function testing who underwent pre-procedural COVID-19 screening test.

**Demographics**	Primary cohort **(N = 586)**	
Age Mean (IQR)	39 (29–52)	
**Frequencies of Gender (N = 586)**	Number (n) and percentage (%)	
Female	235	40%
Male	351	60%
**Frequencies of Nationality (N = 586)**		
Afghan	2	0.3%
Algerian	3	0.5%
American	2	0.3%
Australian	2	0.3%
Bahraini	1	0.2%
Bangladeshi	34	5.8%
British	3	0.5%
Canadian	4	0.7%
Djiboutian	1	0.2%
Egyptian	41	7.0%
Ethiopian	2	0.3%
Filipino	36	6.1%
Gambian	2	0.3%
Indian	75	12.8%
Indonesian	4	0.7%
Iranian	6	1.0%
Iraqi	1	0.2%
Italian	1	0.2%
Jordanian	15	2.6%
Kenyan	12	2.0%
Kuwaiti	2	0.3%
Lebanese	5	0.9%
Maltese	1	0.2%
Moroccan	2	0.3%
Myanmar	1	0.2%
Nepalese	16	2.7%
New Zealander	1	0.2%
Nigerian	1	0.2%
Omani	2	0.3%
Pakistani	20	3.4%
Palestinian	11	1.9%
Portuguese	1	0.2%
Qatari	202	34.4%
Saudi	3	0.5%
Somali	3	0.5%
South African	1	0.2%
Sri Lankan	7	1.2%
Sudanese	26	4.4%
Syrian	9	1.5%
Tunisian	7	1.2%
Turkish	1	0.2%
Ugandan	6	1.0%
Yemeni	11	1.9%

The study found two patients (0.34%) to have a positive COVID-19 RT-PCR on their pre-procedural screening while 4 patients (0.68%) had their results reported as inconclusive. The remaining 580 patients (98.9%) tested negative for COVID-19. The two positive cases were booked to have an elective bronchoscopy (Table 2) to rule out pulmonary tuberculosis (TB) with abnormal imaging features suggestive of pulmonary TB performed for pre-employment screening. Both procedures were postponed. All the patients who were screened before their lung function testing (69%) tested negative. The frequency of procedures performed at our center and their corresponding test results are shown in (Table 2).

**Table 2:** Frequencies of procedures performed and their pre-procedural COVID-19 screening test results.

Procedure (N = 586)	Positive Results	
	N (N = 584)	Y (N = 2)
Bronchoscopy **(N** = **147)**	145	2
EBUS **(N** = **35)**	35	0
Lung Function Test **(N** = **404)**	404	0

A total of 182 (31%) patients were referred for either bronchoscopy or EBUS, or both. These patients were further reviewed for indication of their procedures. The most common indication was to rule out pulmonary TB (79.6%).

## Discussion

Our study sought to assess the rate of positive COVID-19 RT-PCR tests performed on asymptomatic patients with a green status on the contact tracing app (Ehteraz) before electively booked AGPs. Our results showed that the prevalence is relatively low, with 2 patients being positive out of the 586 patients who underwent screening (positivity rate of 0.34%). The rate of positivity also depends on the community prevalence at the time. During the study period, the prevalence of new COVID-19 positive cases varied from a peak of 2355 recorded infections in May 2021 to 235 newly recorded cases at the end of our study [11].

A search of PubMed using Boolean operator strategy for any studies describing pre-procedural screening for COVID-19 in adults in Qatar using the search strategy of (((pre-procedure) OR (bronchoscopy) OR (spirometry)) AND (screening) AND ((COVID-19) OR (SARS-CoV-2)) AND (Qatar)) yielded zero results. Modifying the search strategy outside Qatar, using ((pre-procedure) OR (preprocedure) OR (spirometry) OR (bronchoscopy)) AND (screening) AND ((COVID-19) OR (SARS-CoV-2)) yielded 135 results as of May 2, 2021. Seventeen studies described pre-procedural asymptomatic COVID-19 infection rates in adults detected on PCR-based screening performed before surgeries or various ambulatory procedures. Six of these species were conducted before endoscopies [6,8,9,[12], [13], [14]], with only two studies assessing the COVID-19 positivity rate in patients undergoing bronchoscopic procedures [7,15]. Our positivity rate is similar to Ozturk et al., who found no patients to be positive in asymptomatic pre-procedural screening in their cohort of patients tested [7].

International societies have offered limited guidance on conducting procedures safely. Most of the initial guidelines recommended postponing elective bronchoscopic procedures [16]. For a more urgent indication for the procedure, the common recommendation was to screen all patients for symptoms of COVID-19 and inquire about the history of recent travel or regarding exposure to any individual with symptoms of COVID-19. Additionally, some guidelines suggested performing pre-procedural COVID -19 screening for all patients regardless of symptoms, which is available at the healthcare facility. Irrespective of patients confirmed to be negative before the procedure, strict use of full PPE and infection control policies must be applied (Appendix 3) [17]. These guidelines were based on expert opinion [16]. The local institutional protocol at Hamad Medical Corporation in the early pandemic was to delay elective procedures and to initiate a universal pre-procedural screening protocol.

Additionally, before procedures, temperature screenings, symptom screening, and patient's exposure risk based on the track and trace application ‘Ehteraz’ were also confirmed. The need for pre-procedural testing originally stemmed from the fact that bronchoscopic procedures are aerosol-generating and that asymptomatic carriage of the SARS-CoV-2 virus has been repeatedly demonstrated to be a significant source of transmission leading to the pandemic [18]. However, the true prevalence of encountering such asymptomatic carriage in individuals without risk factors of contracting the virus needs to be investigated.

In our cohort, most bronchoscopic procedures (79.6%) were electively performed to rule out pulmonary TB due to suspicious chest radiography or cross-sectional imaging findings. The State of Qatar has strict guidelines for TB screening as part of the national TB program.

The limitations of universal testing include variable sensitivity of the RT-PCR-based testing kit [10]. It is still unclear whether a positive screening test indicates active COVID-19 infection or prolonged viral shedding, which may not be infectious [19]. Despite these limitations, a documented negative test result may help in reducing the anxiety of HCW performing AGP [9].

All our tested patients were confirmed to have a ‘green’ status on their contact tracing application; hence they had a low risk of exposure to COVID-19. All our patients were also asymptomatic at the time of referral for the procedure, on the day of testing, and the day of the procedure. As the positivity rate is very low in asymptomatic patients, as shown in our study, the risk of infection through aerosol-generating procedures is likely to be very low. HCW carries them out in full PPE irrespective of the screening results. Therefore, the current practice of pre-procedural screening of all asymptomatic patients before bronchoscopy and lung function testing needs to be reassessed to avoid unnecessary costs and delays.

Another aspect to consider is the financial strain of pre-procedural COVID-19 screening. An ideal screening measure should be cost and time-efficient. The cost-effectiveness should take into account the direct and indirect costs to the healthcare system. As per the local infection control policies, during all bronchoscopy procedures, PPE is worn regardless of the COVID-19 status. However, an additional PPE kit is needed for the staff carrying out the pre-procedural test. The average cost of a single complete PPE kit (which included a single-use headcover, goggles, a single-use N95 mask, a single-use isolation gown, two pieces of single-use shoe covers, two pieces of non-sterile disposable gloves, and a face shield) was 75 Qatari Riyals (QAR) roughly 21 United States dollars ($). The price of the PCR test (single kit of swab and the cost to the laboratory for running the test) was quoted to be 180 QAR ($49). The direct costs involved in carrying out PCR testing for COVID-19, the average cost for PPE, and the COVID-19 RT-PCR test were based on the procurement unit's information at Hamad Medical Corporation.

During our study period, 665 asymptomatic patients underwent the PCR test. This equals an additional 119,700 QAR (roughly $33,000) for carrying out the test. This calculation is a conservative estimate as it does not account for the cost of the staffing required for carrying out the test, for laboratory analyses, or dedicated staff liaising the test results with the patient. This estimated cost also does not factor into the indirect patient cost for the transportation to and from the hospital for taking the test. This analysis does not factor in the total cost of lost time for the patient and the healthcare system. For the patient already requiring absenteeism from work to undergo the procedure, a pre-procedural screening test 48–72 h before procedure adds a day to be absent from work, adding to the lost time cost.

Similarly, in an already overburdened virology laboratory, processing these additional screening tests of asymptomatic patients further strains the system. Based on the disease under investigation, patients may also have to undergo multiple pre-procedural COVID-19 screenings adding to further delays. Evidence-based guidelines must therefore be developed for safety and efficacy.

At the time of writing this article, COVID-19 has shown variations in number of cases and different strains of the virus producing recurrent waves of increased positive cases. Considering the ability of the virus to cause such resurgences, the results of this study holds its relevance especially in resource-limited Asian countries whose health-care systems are overburdened in dealing with COVID-19 patients.

In times of resource conservation and reallocation, based on the principle of justice, the case for universal pre-procedural screening for all does not hold up well. High-value care still holds its own in developing and developed healthcare systems.

### Limitations

Our study has expected limitations. Ideally, we would have collected data prospectively from all patients booked for bronchoscopic procedures or lung function testing. However, this was impractical because of unclear impact of not screening patients for COVID-19 and potentially exposing healthcare workers to the virus. The selection of cases labelled as asymptomatic or excluded due to the presence of symptoms was affected by awareness of COVID-19 symptoms particularly early in the pandemic which predisposes to selection bias. Similarly, since the study was conducted early on in the pandemic, there may have been under-reporting by patients of exposure to COVID-19 patients due to which they may be misclassified as low-risk with a ‘green status’ on their contact tracing application. We also recognize that at the time of this study, the testing kits for COVID-19 had variable sensitivities and re-testing them with a repeat RT-PCR after the first negative result may increase the validity of results. However, this was also not feasible in a time where test availability was scarce and virology laboratories were already overburdened. Finally, we acknowledge that the proportion of patients in our cohort who underwent screening who had recently recovered from COVID-19 infection were unknown which may predispose them to have a negative test result on pre-procedural screening.

#### Conclusion

Our results showed that the prevalence of positive COVID-19 test results in asymptomatic patients undergoing bronchoscopic procedures and spirometries is quite low with 2 patients being positive out of the 586 patients who underwent pre-procedural screening with RT-PCR (positivity rate of 0.34%). The authors recommend using pre-procedural symptom and contact screening systems (such as Ehteraz) instead of universal pre-procedural RT-PCR for SARS-CoV-2 for the anticipated resource conservation, decreasing the additional financial burden and reducing the procedural delay. Point of care rapid COVID-19 testing kits with high sensitivity and specificity when available could be another viable alternative.

## Author contribution statement

Ammar Chapra: Performed the experiments; Contributed reagents, materials, analysis tools or data; Wrote the paper.

Zohaib Yousaf: Conceived and designed the experiments; Analyzed and interpreted the data; Contributed reagents, materials, analysis tools or data; Wrote the paper.

Merlin Marry Thomas; Mansoor Hameed: Conceived and designed the experiments; Wrote the paper.

Ahmed Ali A Al-Mohammed; Hisham Abdelaleem A Ahmed: Conceived and designed the experiments; Performed the experiments.

## Funding statement

This work was supported by Qatar National Library (QNL).

## Data availability statement

Data will be made available on request.

## Declaration of interest’s statement

The authors declare no competing interests.

## Declaration of competing interest

The authors declare that they have no known competing financial interests or personal relationships that could have appeared to influence the work reported in this paper.
