# Supplementary material for: Utility of RT-PCR versus electronic track and trace system for pre-procedural COVID-19 screening- a retrospective cohort study
Source: Heliyon. 2023 Apr 11;9(4):e15379. doi: 10.1016/j.heliyon.2023.e15379 (PMC10089668; doi:10.1016/j.heliyon.2023.e15379)
Supplement: Multimedia component 1 [file mmc1.docx]

Appendix 1

New development of the following symptoms within fourteen days before COVID-19 testing was screened for during an initial visit to the Pulmonology outpatient department, on the day of screening test, and the day of the procedure:

- Fever or chills
- New cough
- Shortness of breath or difficulty breathing
- Fatigue
- Muscle or body aches
- Headache
- New loss of taste or smell
- Sore throat
- Congestion or runny nose
- Nausea or vomiting
- Diarrhea

The above process is based on the local protocol provided by the center for disease control (CDC) Qatar and is based on the Center for Disease Control and Prevention protocol. https://www.cdc.gov/coronavirus/2019-ncov/hcp/clinical-guidance-management-patients.html#clinical-presentation. Published 2021, accessed March 30, 2021.

Appendix 2

Process of taking a Nasopharyngeal specimen for COVID-19 RT PCR test.

- Tilt patient’s head back 70 degrees.
- Gently and slowly insert a mini tip swab with a flexible shaft (wire or plastic) through the nostril parallel to the palate (not upwards) until resistance is encountered or the distance is equivalent to that from the ear to the nostril of the patient, indicating contact with the nasopharynx.
- Gently rub and roll the swab.
- Leave the swab in place for several seconds to absorb secretions.
- Slowly remove the swab while rotating it. Specimens can be collected from both sides using the same swab, but it is unnecessary to collect specimens from both sides of the mini tip saturated with fluid from the first collection.
- If a deviated septum or blockage creates difficulty obtaining the specimen from one nostril, use the same swab to obtain the specimen from the other nostril.
- Place swab, tip first, into the transport tube provided.

Process of taking an oropharyngeal specimen for the COVID-19 RT PCR test.

- Insert swab into the posterior pharynx and tonsillar areas.
- Rub swab over both tonsillar pillars and posterior oropharynx and avoid touching the tongue, teeth, and gums.
- Place swab, tip first, into the transport tube provided.

The above processes are based on the local protocol provided by the center for disease control (CDC) Qatar. They are based on the protocol by the Center for Disease Control and Prevention. https://www.cdc.gov/coronavirus/2019-ncov/lab/guidelines-clinical-specimens.html#:~:text=Firmly%20sample%20the%20nasal%20wall,nostril%20using%20the%20same%20swab. Published 2021, accessed March 30, 2021.

Appendix 3

1. Positive:
   - Confirms SARS-CoV-2 viral RNA detection based on meeting the threshold of gene targets based on manufactures recommendations.
2. Negative:
   - Indicates SARS-CoV-2 virus RNA not detected and excludes a diagnosis of COVID-19.
3. Inconclusive:
   - Where gene targets do not meet the definition of either positive or negative.
   - As per the local guidelines from CDC Qatar:
     1. For highly suspected patients with clinical and radiological findings, or patients being tested following contact with a confirmed case of COVID-19, or being tested as part of an outbreak, then an inconclusive result should be repeated after 24 hours.
     2. An inconclusive result should be interpreted as negative for follow-up in an individual with confirmed COVID-19 infection with resolved symptoms.
     3. For testing routine asymptomatic patients, for example, pre-procedure, an inconclusive result should be interpreted as negative.

Appendix 4

Infection control measures taken during bronchoscopic procedures or lung function testing:

- HCP in the room should wear an N95 or equivalent or higher-level respirator, eye protection, gloves, and a gown.
- The number of HCP present during the procedure should be limited to only those essential for patient care and procedure support. Visitors should not be present for the procedure.
- If possible, aerosol Generating Procedures (AGPs) should occur in Airborne Infection Isolation Rooms (AIIRs).
- Clean and disinfect procedure room surfaces promptly, as described in the section on environmental infection control below.

The above process is based on the local protocol provided by the center for disease control (CDC) Qatar and is based on the Centers for Disease Control and Prevention protocol. https://www.cdc.gov/coronavirus/2019-ncov/hcp/infection-control-recommendations.html. Published 2021. Accessed March 30, 2021.
